# Rutin Modulates MAPK Pathway Differently from Quercetin in Angiotensin II-Induced H9c2 Cardiomyocyte Hypertrophy

**DOI:** 10.3390/ijms22105063

**Published:** 2021-05-11

**Authors:** Hawa Nordin Siti, Juriyati Jalil, Ahmad Yusof Asmadi, Yusof Kamisah

**Affiliations:** 1 Department of Pharmacology, Faculty of Medicine, Universiti Kebangsaan Malaysia, Kuala Lumpur 56000, Malaysia; hawanordin@gmail.com; 2 Department of Basic Medical Sciences, Faculty of Medicine, Universiti Sultan Zainal Abidin, Kuala Terengganu 20400, Malaysia; 3 Drug and Herbal Research Centre, Faculty of Pharmacy, Universiti Kebangsaan Malaysia, Kuala Lumpur 50300, Malaysia; juriyatijalil@ukm.edu.my; 4 Unit of Pharmacology, Faculty of Medicine and Defence Health, Universiti Pertahanan Nasional Malaysia, Kuala Lumpur 57000, Malaysia; draayusof@gmail.com

**Keywords:** flavonoids, cardiomyocyte hypertrophy, ERK, JNK, p38 kinase, inducible nitric oxide synthase

## Abstract

Rutin is a flavonoid with antioxidant property. It has been shown to exert cardioprotection against cardiomyocyte hypertrophy. However, studies regarding its antihypertrophic property are still lacking, whether it demonstrates similar antihypertrophic effect to its metabolite, quercetin. Hence, this study aimed to investigate the effects of both flavonoids on oxidative stress and mitogen-activated protein kinase (MAPK) pathway in H9c2 cardiomyocytes that were exposed to angiotensin II (Ang II) to induce hypertrophy. Cardiomyocytes were exposed to Ang II (600 nM) with or without quercetin (331 μM) or rutin (50 μM) for 24 h. A group given vehicle served as the control. The concentration of the flavonoids was chosen based on the reported effective concentration to reduce cell hypertrophy or cardiac injury in H9c2 cells. Exposure to Ang II increased cell surface area, intracellular superoxide anion level, NADPH oxidase and inducible nitric oxide synthase activities, and reduced cellular superoxide dismutase activity and nitrite level, which were similarly reversed by both rutin and quercetin. Rutin had no significant effects on phosphorylated proteins of extracellular signal-related kinases (ERK1/2) and p38 but downregulated phosphorylated c-Jun N-terminal kinases (JNK1/2), which were induced by Ang II. Quercetin, on the other hand, had significantly downregulated the phosphorylated proteins of ERK1/2, p38, and JNK1/2. The quercetin inhibitory effect on JNK1/2 was stronger than the rutin. In conclusion, both flavonoids afford similar protective effects against Ang II-induced cardiomyocyte hypertrophy, but they differently modulate MAPK pathway.

## 1. Introduction

Cardiac hypertrophy is commonly manifested in heart failure [1]. Neurohormonal system activation is involved in the development of cardiac hypertrophy, with angiotensin II (Ang II) being the key player in inducing cardiomyocyte hypertrophy. Cardiomyocytes respond to Ang II by initiating several cascades that lead to hypertrophy [2]. The binding of Ang II on angiotensin type 1 receptor (AT1R), a G-protein-coupled receptor (GPCRs), causes the dissociation of different G-proteins that activate several signaling kinases and phosphatases including mitogen-activated protein kinases (MAPKs) [2]. MAPKs are a group of serine and threonine protein kinases that are encoded by multiple genes. Extracellular signal-related kinases (ERK1/2), c-Jun N-terminal kinases (JNK1/2), and p38 kinase (p38) are three MAPKs that have been reported to be involved in cardiac hypertrophy [3]. Activation of AT1R by Ang II also activates NADPH oxidase that increases the generation of reactive oxygen species (ROS), particularly superoxide anion [4]. Superoxide dismutase (SOD) converts the anion to less reactive molecules. An elevation of inducible nitric oxide synthase (iNOS) is also observed in cardiomyocyte hypertrophy [5].

Many in vitro and in vivo studies have extensively shown the ability of quercetin, a flavonoid to attenuate ventricular hypertrophy [6,7]. However, studies regarding the effects of rutin, another type of flavonoid on cardiac hypertrophy, in particular, are far less extensive than that of its metabolite, quercetin. Rutin is a glycoside (rutinose) of the quercetin [8] and also known as rutoside, rutinum, vitamin P, sophorin, and quercetin-3-O-rutinoside. It is diversely found in plants like tea and citrus fruits [9,10]. While, quercetin (3,3′,4′,5,7-pentahydroxyflavone) is found in many plants such as apples, onions, citrus fruits, berries, and red grapes [8]. Both flavonoids show potent antioxidant [11,12] and anti-inflammatory [13,14] properties. Mediterranean diet is rich in flavonoids. The intake of the diet is reported to prevent cardiovascular diseases [15]. Currently, studies have been conducted to investigate the potential anticancer [16,17,18,19] and antiviral [20,21,22] properties of quercetin and rutin. In addition, combination therapy of quercetin and vitamin C has been reported to be beneficial for the prevention and early treatment of severe acute respiratory syndrome coronavirus-2 (SARS-CoV-2 or COVID-19) [23].

Based on the current research development of the flavonoids on cardiomyocyte hypertrophy, it is not clear whether rutin demonstrates similar effects to quercetin. Hence, this study aimed to investigate the effects of the flavonoids on Ang II/ROS/nitric oxide (NO) axis, and MAPK pathway in Ang II-induced hypertrophied cardiomyocytes.

## 2. Results

### 2.1. Ang II Increased Cardiomyocytes Cell Surface Area and B-Type Natriuretic Peptide Level

**Figure 1:**
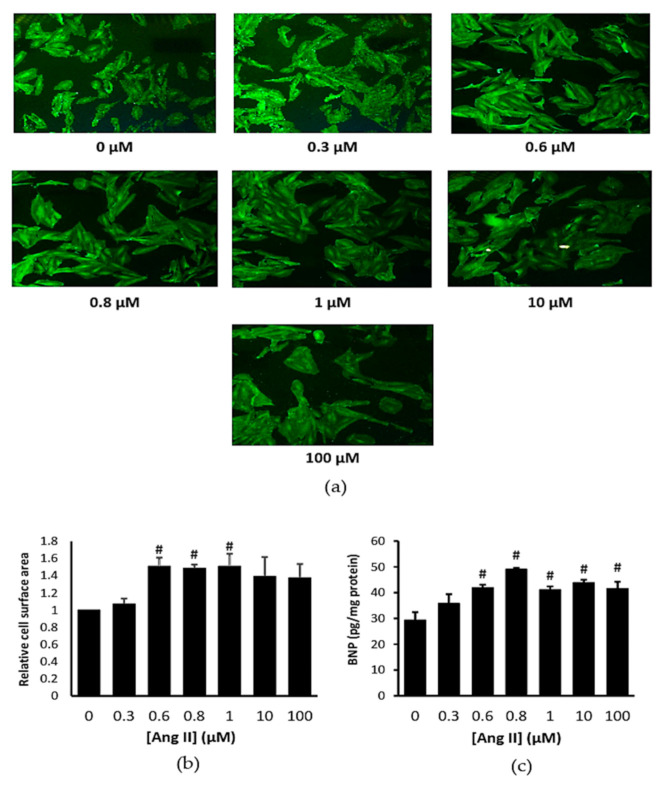
(**a**) Representative immunofluorescent staining (magnification ×100), (**b**) cell surface area, and (**c**) B-type natriuretic peptide (BNP) level in H9c2 cells exposed to Ang II at various concentrations for 24 h. ^#^ *p* < 0.05 versus control group. Data were analyzed by a one-way ANOVA followed by a Tukey post hoc test and are represented as the mean ± SEM (*n* = 3).

Representative image of the treated cells with Ang II are presented in Figure 1a. Ang II had significantly increased cell surface area compared to control at the concentrations of 0.6, 0.8, and 1 μM (*p* < 0.05) (Figure 1b). Ang II also increased the cellular B-type natriuretic peptide (BNP) level starting from 0.6 µM concentrations (*p* < 0.05) (Figure 1c), confirming that Ang II successfully induced cardiomyocyte hypertrophy. Based on these data, we chose 0.6 µM (600 nM) Ang II for the subsequent experiments.

### 2.2. Rutin and Quercetin Inhibited Ang II-Induced Cardiomyocyte Hypertrophy and BNP Level

**Figure 2:**
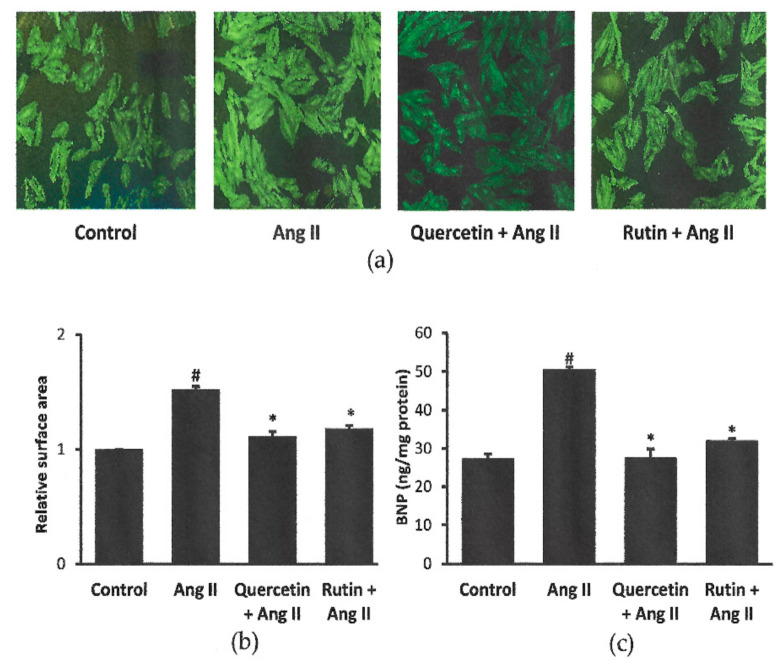
Effects of quercetin (331 μM) and rutin (50 µM) on H9c2 cell size shown by (**a**) representative immunofluorescent staining (magnification ×100), (**b**) cell surface area and (**c**) cellular BNP level after 24 h co-treatment with Ang II (600 nM). ^#^ *p* < 0.05 vs. control group, * *p* < 0.05 vs. Ang II group. Data were analyzed by a one-way ANOVA followed by a Tukey post hoc test and are represented as the mean ± SEM (*n* = 3).

Representative images of the cells coexposed to Ang II with or without quercetin or rutin are shown in Figure 2a. Cells exposed to Ang II alone had significantly bigger cell size (1.52 ± 0.02 ratio of control cells) and higher cellular BNP level (50.49 ± 0.67 ng/mg protein) than the control cells (27.29 ± 1.20 ng/mg protein). Treatment with rutin or quercetin had significantly inhibited the induction of hypertrophy by Ang II in H9c2 cells, in terms of cell size and BNP level (Figure 2b,c). There was no significant difference in cell size and cellular BNP level between rutin, quercetin, and control groups.

### 2.3. Rutin and Quercetin Inhibited Ang II-Induced Intracellular Superoxide, NADPH Oxidase, and SOD Activities

**Figure 3:**
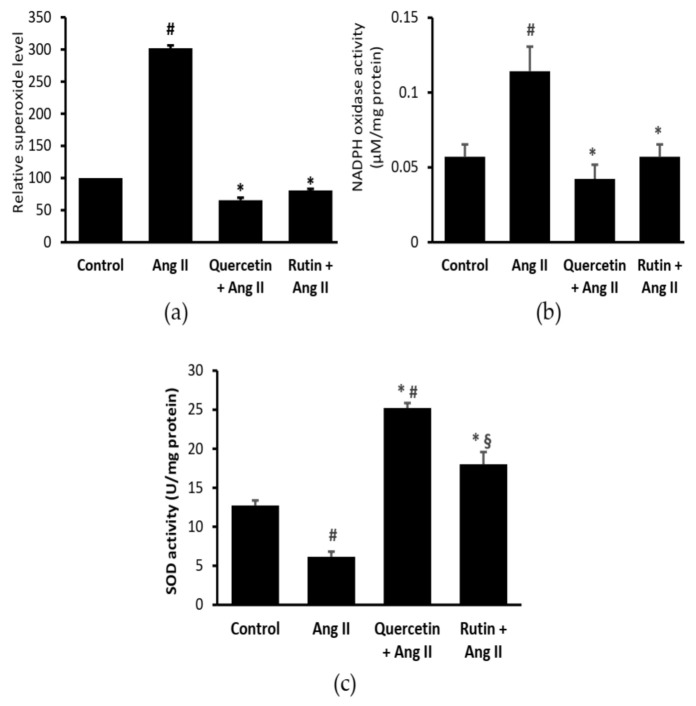
(**a**) Intracellular superoxide, (**b**) cellular NADPH oxidase, and (**c**) superoxide dismutase (SOD) activities in cardiomyocytes coexposed to Ang II and quercetin (331 µM) or rutin (50 µM) for 24 h. ^#^ *p* < 0.05 vs. control group, * *p* < 0.05 vs. Ang II group, ^§^ *p* < 0.05 vs. quercetin + Ang II. Data were analyzed by a one-way ANOVA followed by a Tukey post hoc test and are represented as the mean ± SEM (*n* = 3).

Ang II significantly elevated the intracellular superoxide level (Figure 3a) and NADPH oxidase activity (Figure 3b), and reduced SOD activity (Figure 3c) in H9c2 cells. Both rutin and quercetin treatments similarly reduced the Ang II-induced superoxide generation and NADPH oxidase activity. Both flavonoids also reversed the effects of Ang II on SOD activity, but the effect of quercetin was more prominent.

### 2.4. Quercetin and Rutin Reduced Cellular iNOS and NO Levels

**Figure 4:**
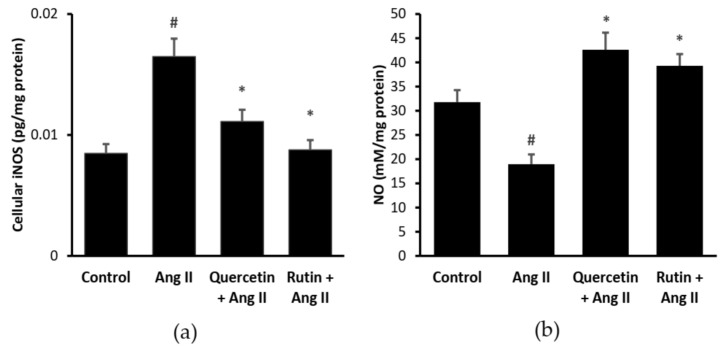
(**a**) Cellular inducible nitric oxide synthase (iNOS) and (b) NO levels in different groups. ^#^ *p <* 0.05 vs. control group, * *p <* 0.05 vs. Ang II alone treated cells. Data were analyzed by a one-way ANOVA followed by a Tukey post hoc test and are represented as the mean ± SEM (*n* = 3).

Figure 4a shows that Ang II significantly increased cellular iNOS level (0.016 ± 0.001 pg/mg protein, *p* < 0.05) in H9c2 cells than that of the control (0.008 ± 0.001 pg/mg protein). iNOS level was significantly reduced (*p* < 0.05) by the treatments of rutin (0.008 ± 0.001 pg/mg protein) and quercetin (0.011 ± 0.001 pg/mg protein). There was no significant difference in the iNOS level between the rutin, quercetin, and control cells.

Ang II had significantly reduced cellular NO level (18.96 ± 2.01 mM/mg protein, *p* < 0.05) in H9c2 cells compared with the control (31.79 ± 2.48 mM/mg protein) (Figure 4b). Rutin (39.31 ± 2.41 mM/mg protein) and quercetin (42.61 ± 3.54 mM/mg protein) managed to reverse the detrimental effect of Ang II (*p* < 0.05). There was no significant difference in NO level between the rutin, quercetin, and control groups.

### 2.5. Quercetin and Rutin Affects MAPK Activation Differently

**Figure 5:**
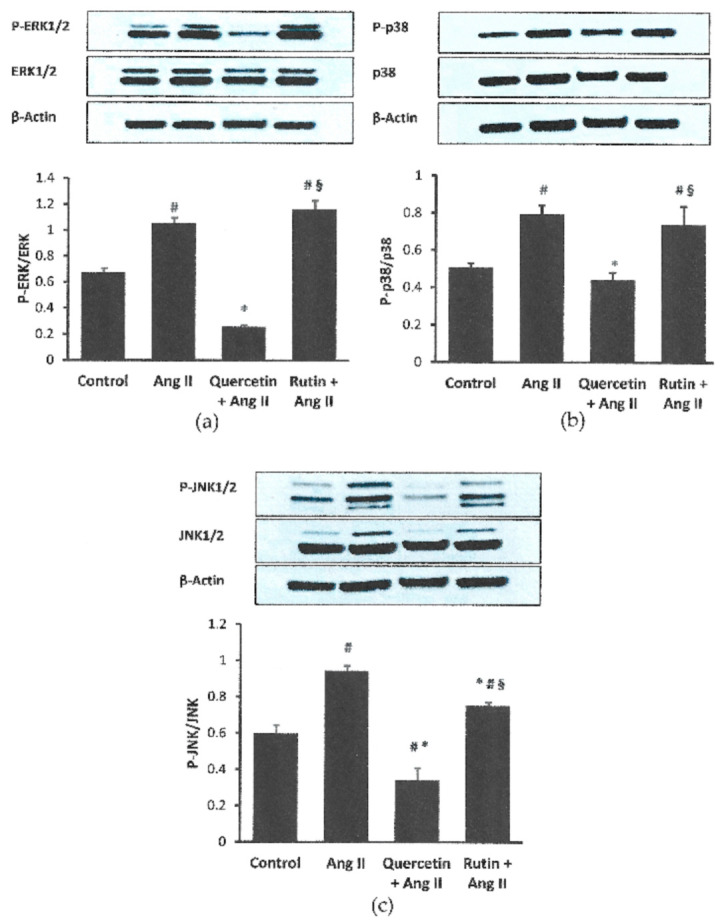
Effects of quercetin (331 μM) and rutin (50 µM) total on the ratio of phosphorylated: total (**a**) ERK1/2, (**b**) p38, and (**c**) JNK1/2 in H9c2 cells exposed to Ang II (600 nM) for 24 h. Data were analyzed by a one way ANOVA followed by a Tukey post hoc test and are represented as the mean ± SEM (*n* = 3). ^#^ *p <* 0.05 vs. control, * *p <* 0.05 vs. Ang II; ^§^ *p <* 0.05 vs. quercetin + Ang II.

Exposure to Ang II increased the expression of phosphorylated ERK1/2 but no effect on total ERK1/2, resulting in an elevated ratio of the phosphorylated and total ERK1/2 in H9c2 cells (Figure 5a). Cotreatment of quercetin had significantly blocked the phosphorylation of the ERK1/2, but no effect on the total expression. Hence, the ratio of the phosphorylated and total was decreased. Rutin, on the other hand, had no effect on this protein.

The phosphorylated p38 protein was significantly increased in H9c2 cells in response to Ang II exposure (*p <* 0.05) compared with the control (Figure 5b). Coincubation with quercetin had significantly reduced the phosphorylated p38, but did not affect the total expression. The ratio of the proteins was significantly reduced in the quercetin-treated group. Rutin coincubation had no significant effect on this protein in cells exposed to Ang II.

Ang II had augmented phosphorylated and total JNK1/2 proteins in the cardiomyocytes (Figure 5c). Rutin reduced the phosphorylated protein, while quercetin reduced both phosphorylated and total proteins in the cells exposed to Ang II. The ratio of the phosphorylated and total JNK1/2 was significantly lower in the quercetin- and rutin-treated groups. The phosphorylated and its ratio to total protein expressions in the rutin-treated group were significantly higher than the quercetin-treated group (*p* < 0.05).

## 3. Discussion

The present study demonstrated that Ang II caused an imbalance in ROS/NO axis, which partly contributed to cardiomyocyte hypertrophy, evidenced by increases in cell surface area and BNP level. Ang II elicited a series of events involving (1) an increase in intracellular superoxide production associated with increased NADPH oxidase enzyme activity, and decreased SOD activity; (2) an increase in iNOS level; and following (3) a decrease in nitrite level in cardiomyocytes. The elevated levels of intracellular superoxide and NADPH oxidase activity, along with a reduction in SOD in the Ang II-induced hypertrophied cells confirmed the development of oxidative stress that took place during hypertrophy. Our data showed that both rutin and quercetin had antioxidant properties as evidenced by their similar abilities to inhibit intracellular superoxide level. NADPH oxidase is an important source of superoxide in the cardiovascular system [24], via activation of AT1R by Ang II [25]. In our study, the inhibitory effects of rutin and quercetin on Ang II-induced NADPH oxidase most likely contributed to the reduction in the superoxide level. This was associated with the elevated endogenous SOD enzyme activity by these compounds, leading to reduced cell surface area, indicating prevention of cardiomyocyte hypertrophy development. Flavonoids have been reported to increase SOD activity by binding their hydroxyl group to the SOD molecules, thereby stabilizing them [26,27]. Both compounds are able to scavenge superoxide and peroxynitrite radicals [12,28,29]. However, rutin had a lower ability than quercetin in augmenting SOD activity, which could be due to the difference in their structures, which warrants further investigation.

Other than superoxide production by NADPH oxidase, ROS can also be generated through upregulation of iNOS, in addition to NO synthesis [11]. Ang II has been known to cause cardiac inflammation by inducing iNOS [30], as observed in the present study. Even though NO is a product of iNOS activity, it was noted to be reduced by the Ang II exposure in this study. It was most likely that the diminished level of the NO could be due to increased interaction between it and the superoxide, producing more peroxynitrite radicals [31]. The reversal effect of both rutin and quercetin on the iNOS and NO indicated their anti-inflammatory property. Similar effects of quercetin and rutin against the elevated iNOS and NO levels were also reported by other studies [14,32,33].

In other studies, quercetin was used in the range of 20−100 µM in primary neonatal rat ventricular cardiomyocytes [6,34,35]. However, we chose 331 µM following a study by Yan et al. [36], which used a similar model to ours—Ang II-induced hypertrophy in H9c2 cells—that showed the best cardioprotective effect and appeared to be non-toxic to the cells. The selection was based on a report by Peter et al. [37] that different cells responded differently to the same compound even at a similar concentration. While for rutin, to the best of our knowledge, there was no other study that investigated the effects of rutin on cardiomyocyte hypertrophy in H9c2 cells. Therefore, its concentration (50 µM) in H9c2 cells exposed to pirarubicin-induced injury [38] was selected.

Our data suggest that the cardioprotective and antihypertrophic effects of rutin and quercetin might be associated with their effects on ROS/NO axis. ROS may be involved in more than one intracellular signaling pathways [39]. In fact, the production of ROS is also linked to inducers of hypertrophy such as the MAPKs [39,40]. There is substantial evidence indicating excessive production of ROS can activate the MAPK pathways [39,41], which play a significant role in the pathogenesis of cardiomyocyte hypertrophy [3].

Ang II is one of the stimuli that can activate ERK1/2, JNK, and p38 [42,43,44]. When exposed to extracellular stimuli like Ang II, signals will be transduced through a GPCR. It will further lead to sequential phosphorylation events that activate the MAPK cascade. Phosphorylation of ERK1/2, JNK1/2, and p38 MAPKs at the threonine/tyrosine residues results in a conformational change. The activated MAPKs are then translocated into the nucleus, where they catalyze the phosphorylation of several transcription factors, which control the expression of targeted genes [45,46]. BNP is one of the important products of downstream hypertrophy gene stimulation by Ang II [47,48]. Its level has been used as a diagnostic and prognostic marker in patients with heart failure risk and ventricular hypertrophy [48,49]. Based on our data, rutin and quercetin were able to prevent Ang II-induced cardiomyocytes hypertrophy as observed by the reduction in cell size area and BNP level.

We believe that the cardioprotective effects of quercetin most possibly involves modulation of Ang II-induced MAPK activation, as quercetin significantly reduced the upregulation of phosphorylation of ERK1/2, JNK1/2, and p38 relative to its total expression, suggestive that the protective action of quercetin obviously by suppressing the activation of ERK1/2, JNK1/2, and p38. It seems that quercetin could become a potential compound for the development of specific inhibitors for ERK1/2, p38, and JNK1/2. Although both quercetin and rutin seemed to be targeting the ROS/NO axis, they acted differently on the MAPK pathway. To the best of our knowledge, this was the first study to investigate the effect of rutin on ERK1/2, JNK1/2, and p38 using the Ang II-induced H9c2 hypertrophy model. Unlike its metabolite quercetin, rutin did not suppress the activation of ERK1/2 and p38, which suggested that the effects of the flavonoids on MAPK pathway might be relying on their structures rather than radical scavenging per se. Unlike quercetin, the inhibitory effects of rutin on the ERK1/2 and p38 phosphorylation were almost absent, confirming that rutin did not possess an intrinsic ability to inhibit phosphorylation of ERK1/2 and p38. The explanation for rutin that had only affected the activation of JNK1/2 was unclear. It could be owing to selective inhibition of the upstream kinases of JNK1/2 signaling cascade by the rutin. Indeed, studies on the effects of rutin on cardiac hypertrophy are still lacking. Chu et al. [50] found that rutin from buckwheat inhibited Ang II-induced cardiomyocyte hypertrophy via inhibition of calcineurin-dependent signal pathway. Rutin may modulate cardiomyocyte hypertrophy through other signaling pathways. A limitation in the present study was the absence of the flavonoid groups not treated with Ang II. Accordingly, the effects of quercetin and rutin alone on the measured parameters could not be ascertained. However, previous studies had reported comparable effects of quercetin on iNOS and p38 expressions, NO level, ROS production [40], protein, and collagen 1 content to the control [34,35] in cardiomyocytes, suggestive of no significant effect of the compound in the absence of a stressor.

Li et al. [51] found that rutin exerted a lesser cardioprotective effect than its metabolite, quercetin on isoproterenol-induced cardiac fibrosis in the rats, while Mladenka et al. [52] found that intravenous administration of rutin did not protect against isoprenaline-induced cardiotoxicity. We suggest that the beneficial effect of rutin against Ang II induced-oxidative stress observed in our study was directly attributable to the parent compound itself, but the modulation of MAPKs was probably better by its metabolites like quercetin. In vivo, it is metabolized by intestinal bacteria to several metabolites, importantly quercetin and isorhamnetin [53]. Therefore, it may give different outcomes when studied in in vitro and in vivo, and via different routes. Current research focuses on phosphodiesterase 10A as a new therapeutic target, which expression is elevated in cardiac hypertrophy [54]. Both quercetin and rutin have been shown to possess an inhibitory effect on phosphodiesterase [55,56]. Hence, for future research perspectives on these flavonoids, their potential as a phosphodiesterase inhibitor could be further studied.

## 4. Materials and Methods

### 4.1. Culture of Myocardial H9c2 Cell Line

H9c2 cells were acquired from the American Type Culture Collection (ATCC, Rockville, MD, USA) and grown in Dulbecco’s modified Eagle medium (DMEM) (Gibco, Grand Island, NY, USA), added with 10% fetal bovine serum (FBS) (Sigma-Aldrich, St. Louis, MO, USA) containing 100 U/mL penicillin G, 100 μg/mL streptomycin, in an incubator with humidified air containing 5% CO_2_ at 37 °C. The culture medium was replaced on alternate days. Cells of passages 5–7 were utilized for each experiment. The cells were grown to 70% confluency, then serum-starved for 24 h before treatment [36].

### 4.2. Concentration-Response Studies of Ang II on Cardiomyocytes

Ang II (Sigma-Aldrich, St. Louis, MO, USA) was used to induce H9c2 hypertrophy, as previously established [57]. In brief, H9c2 cells were seeded in 96-well plate (1 × 10^4^ cells/well) or 8-well chamber slides (1 × 10^4^ cells/well) or 10 mm^2^ culture dishes (1.5 × 10^5^ cells/dish) and grown to 70% confluency. The cells were then incubated with different concentrations of Ang II (0.3–500 μM) for 24 h. The cells were later collected for analysis (cell surface area and cellular BNP level). An optimal concentration of Ang II was chosen to induce cardiomyocyte hypertrophy in the subsequent experiments.

### 4.3. Experimental Groups

In the subsequent experiments, Ang II at 600 nM was used to induce cell hypertrophy. H9c2 cells were randomly allocated into four different groups: Control, Ang II, Ang II + quercetin (331 μM) [36], and Ang II + rutin (50 µM) [38]. The cells were treated with Ang II alone or in combination with either quercetin or rutin for 24 h. The final DMSO concentration used as a vehicle for quercetin and rutin was less than 0.1% [19]. The concentration of the flavonoids was selected according to the effective concentration reported to reduce cell hypertrophy and cardiac injury in H9c2 cells [36,38]. Both quercetin (Q4951) and rutin (R5143) were commercially obtained from Sigma-Aldrich (St. Louis, MO, USA).

### 4.4. Cell Size Measurement

Cell size was measured according to an established method [58]. Briefly, treated cardiomyocytes were fixed with 4% paraformaldehyde for 10 min before being permeabilized with 0.2% Triton-X for 10 min. Then, the cells were blocked with 10% FBS for an hour at 37 °C, before being incubated with mouse antisarcomeric α-actinin monoclonal primary antibody (Cat. No.: ab9465; Abcam, Cambridge, MA, USA; 1:200) at 4 °C overnight, followed by the Alexa Fluor 488-conjugated rabbit antimouse polyclonal secondary antibody (Cat. No.: A-11059, Invitrogen, Waltham, MA, USA; 1:200) for an hour in the dark, at room temperature. The cells were then washed, and the staining was observed under a fluorescence microscope (Olympus Optical, Tokyo, Japan). ImageJ software was used to measure the cardiomyocyte surface area. The size of at least 60 cells (actinin-positive cells) per chamber for each experimental group was measured by a blinded assessor and expressed as a ratio to control cells.

### 4.5. Cellular BNP Level and iNOS Activity

After respective treatments, cell lysates were collected and assayed for BNP and iNOS level using BNP (E-EL-R0126; Elabscience, Houston, TX, USA) and iNOS (E-EL-R0520; Elabscience, Houston, TX, USA) kits, respectively following the manufacturer’s protocol. Briefly, the samples and biotinylated detection antibody working solution into were pipetted micro-ELISA wells that were precoated with BNP or rat NOS2/iNOS-specific antibody and incubated at 37 °C. After the removal of unbound samples or excess conjugate, avidin-horseradish peroxidase (HRP) conjugate and substrate solutions were added, and incubated after each addition. These reactions developed blue in color, which turned yellow after addition of the stop solution. The absorbance of the reactions was read at 450 nm, and the BNP level and activity of the iNOS were calculated against their respective standards.

### 4.6. Intracellular Superoxide Detection

The detection of superoxide in living cells was done using a commercial kit (ROS-ID^®^ Total ROS/Superoxide Detection Kit, ENZ-51010, Enzo Life Science Ltd., NY, USA). The cardiomyocytes were seeded at cell density 1 × 10^4^ cells/ well in a 96-well plate. After treatment, the cells were incubated with superoxide detection dye (100 µL/well) for an hour in a humidified incubator (37 °C, 5% CO_2_). The absorbance was read using a fluorescence microplate reader (EnSpire^®^ Multimode Plate Reader, PerkinElmer, Inc., Hopkinton, MA, USA) equipped with an orange filter (λEx = 550 nm and λEm = 610 nm). 

### 4.7. NADPH Oxidase and SOD Activities

NADPH oxidase activity was measured in total cell lysate (50 μg protein/sample), according to the method described by Mustapha et al. [59]. The enzyme activity was calculated from the difference between absorbance of samples at 120 min with or without diphenyleneiodonium (DPI), an NADPH oxidase inhibitor, using an extinction coefficient of 21 mmol/L/cm and expressed in nmol/mg protein. 

The activity of SOD was assayed using an established method [60], based on its capacity to inhibit the reduction of nitro blue tetrazolium (NBT) by superoxide. The reduction of NBT was measured at 550 nm (EnSpire^®^ Multimode Plate Reader, Perkin Elmer, Inc., Hopkinton, MA, USA). One enzyme unit is defined as the amount of enzyme to produce half-maximal inhibition of NBT reduction. The results were expressed in U/mg protein.

### 4.8. Cellular Nitrite

Nitrite is a stable metabolite that represents the level of NO. It was measured following a recently described method [61]. Briefly, 50 μL sample cell lysates were loaded into a microtiter plate, mixed with 50 µL of modified Griess reagent (Sigma Aldrich, St. Louis, MO, USA), and then incubated in the dark at room temperature for 15 min. The absorbance was read at 540 nm wavelength (EnSpire^®^ Multimode Plate Reader, PerkinElmer, Inc., Hopkinton, MA, USA). The NO concentrations were estimated against sodium nitrite curve (Sigma-Aldrich, St. Louis, MO, USA). 

### 4.9. Western Blot Analysis

After treatment, proteins from each group were obtained with radio immunoprecipitation assay (RIPA) lysis buffer (Sigma-Aldrich, St. Louis, MO, USA) containing the protease inhibitor cocktail (Roche, Basel, Switzerland) and phosphatase inhibitors consist of 10 mM sodium fluoride (R&M Chemicals, Essex, UK), 10 mM sodium orthophosphate (R&M Chemicals, Essex, UK), and 1 mM sodium orthovanadate (Sigma Aldrich, St. Louis, MO, USA). The proteins were subjected to electrophoresis using a sodium dodecyl sulfate–polyacrylamide gel electrophoresis (10%, SDS-PAGE) and transferred to polyvinylidene difluoride (PVDF) membrane (Bio-Rad Laboratories, Hercules, CA, USA). The membranes were blocked with 5% *w/v* bovine serum albumin in 1% Tris-buffered saline and 0.1% Tween 20 (Bio-Rad Laboratories, Hercules, CA, USA) for an hour, before being incubated with primary antibodies against phospho-ERK1/2 (#4377; rabbit polyclonal; 1:1000; Cell Signaling Technology, Danvers, MA, USA), phospho-JNK1/2 (#4668; rabbit monoclonal; 1:1000; Cell Signaling Technology, Danvers, MA, USA), p-p38 (sc-166182; mouse monoclonal; 1:500 dilution; Santa Cruz Biotechnology, St Dallas, TX, USA), ERK1/2 (#4695; rabbit monoclonal; 1:1000; Cell Signaling Technology, Danvers, MA, USA), JNK1/2 (#9252; rabbit polyclonal; 1:1000; Cell Signaling Technology, Danvers, MA, USA), and p38 (#8690; rabbit monoclonal; 1:1000; Cell Signaling Technology, Danvers, MA, USA) at 4 °C overnight. β-Actin (sc-47778; mouse monoclonal; 1:500; Santa Cruz Biotechnology, St Dallas, TX, USA) was used as a loading control. After a thorough wash, the membranes were incubated with horseradish peroxidase-conjugated IgG (sc516102; anti-mouse, 1:2000; Santa Cruz Biotechnology, St. Dallas, TX, USA) antibody for 1 h at room temperature. Blots were then developed with enhanced chemiluminescence developing solution (ECL; Bio-Rad, Hercules, CA, USA) and visualized using the gel documentation system and quantified using ImageJ software.

### 4.10. Statistical Analysis

Data were expressed as mean ± standard error (SEM) with at least three independent triplicate experiments. The Shapiro–Wilk test was used for the normality test. The results were analyzed using a one-way analysis of variance (ANOVA) followed by a Tukey post hoc test, using SPSS v24.0 software. The accepted statistically significant value was *p <* 0.05.

## 5. Conclusions

Our findings suggest that both rutin and quercetin had similarly prevented Ang II-induced cardiomyocyte hypertrophy by blunting the ROS/NO axis. Rutin had weaker inhibitory effects on JNK1/2 activation than quercetin without affecting ERK1/2 and p38 expressions. Rutin may manifest its protective effects via other signaling pathways, which warrants further investigation.
